# Physiologically based pharmacokinetic (PBPK) modeling and simulation in drug discovery and development

**DOI:** 10.5599/admet.667

**Published:** 2019-02-23

**Authors:** Abdul Naveed Shaik, Ansar Ali Khan, ADMET & DMPK

**Affiliations:** 1 Simulations Plus Inc., Lancaster, CA. USA; 2 GVK Biosciences. Hyderabad, TS. India

Physiologically based pharmacokinetic (PBPK) modeling is a mechanistic or physiology based mathematical modeling technique which integrates the knowledge from both drug-based properties including physiochemical and biopharmaceutical properties and system based or physiological properties to generate a model for predicting the absorption, distribution, metabolism and excretion (ADME) of a drug as well as pharmacokinetic behavior of a drug in preclinical species and humans. Even though PBPK models can provide mechanistic insights, it was seldom used for long time due to the lack of high-performance computing systems. With the advent of high-performance computing facilities, multiple differential equations can be solved at a time. Development of commercial PBPK software such as PK-Sim (Bayer Technology Services), GastroPlus (Simulations Plus Inc.), SimCYP (Certara), SimBiology (MathWorks) etc. has resulted in increased use of PBPK in drug development. PBPK models can be used in different phases of drug development including the first in human (FIH) dose prediction from preclinical data. In this phase data generated from *in vitro* experiments as well as data from *in silico* predictions can be integrated to develop model which can then be used to predict FIH dose range. This is referred as the bottoms up approach. On the other hand, in the top down approach, clinical data can be used to develop and refine the PBPK model. Moreover, PBPK model can be used to evaluate drug safety and toxicity, as well as the prediction of drug exposure in different conditions or populations studying the effect of age, gender, ethnicity disease state etc. These models are also commonly used in the evaluation of drug-drug interaction (DDI) to provide insights into any undesirable issues of the investigative new drug (IND) with other marketed drugs.

Recent advancement in PBPK modeling and the understanding of metabolizing enzymes, drug transporters and genetic make-up of different races have led to an increase in the use of PBPK models in drug discovery and development. These models can provide a simple *in vitro in vivo* correlation (IVIVC) based on the thorough *in vitro* data from drug metabolism [1-3] and drug transporter studies [2, 4]. PBPK is very useful in modelling of: a) absorption where the model takes into consideration the solubility, dissolution, and permeability both passive and active with the involvement of transporters, gut extraction through metabolism; b) distribution where physiological parameters and compound related parameters are taken into consideration and various equations can be applied to calculate the tissue partition coefficient (*K*_p_) for perfusion rate limited or permeability limited situations; c) metabolism where data from *in vitro* sources including recombinant enzymes, liver microsomes and hepatocytes and transporter data from hepatocytes or over expressed cell lines can be used to inform the rate and extend of *in vivo* metabolism; d) excretion through biliary, renal routes can be easily modelled using PBPK models.

There is an increase in the acceptability of PBPK models in drug submission package by USFDA, EMA and PMDA as evident by the fact that the number of IND applications as well as submissions with DDIs and in special populations including paediatrics that involved the use of PBPK have increased significantly in the recent years by regulatory agencies. In particular, these regulatory agencies have issued guidance for application and use of PBPK models at various stages of drug development including, investigational new drug applications (INDs), new drug applications (NDAs), biologics license applications (BLAs), or abbreviated new drug applications (ANDAs) etc. With the increased use of pharmacodynamics (PD) with PBPK models (PBPK-PD) where simple PD models can be linked to PBPK model, it is anticipated that PBPK model-based drug development is going to be very popular in pharmaceutical research. In the foreseeable future, we anticipate that more pharmaceutical companies and academicians are going to adopt PBPK modeling in their research and drug development projects.

With the increase in the number of research publication utilizing PBPK from industry, academia and regulatory authorities alike and with the success of prior special issues focused on various stages of drug development we devoted a special issue on “Physiologically based pharmacokinetic (PBPK) modeling and simulation in drug discovery and development”. We received four original articles and a review article for this special issue. These articles covered varied areas including “Building “in-house” PBPK modeling tools for oral drug administration from literature information” [5] where the researchers have shown how to develop in-house PBPK tools from species-related physiological information available in the literature and a limited number of drug specific parameters and validated the in-house PBPK tools with 25 compounds. Another research article entitled “A physiologically-based pharmacokinetic model of oseltamivir phosphate and its carboxylate metabolite for rats and humans” [6] where the model was built with both the parent oseltamivir phosphate and its carboxylate metabolite. The model built in healthy subjects has been applied in disease-subjects as well as in subjects with liver or renal impairment. One other research article was titled "Physiologically-based pharmacokinetic simulations in pharmacotherapy: selection of the optimal administration route for exogenous melatonin" [7] where the authors showed the significance of PBPK model in dose optimization and selection of route of administration. One article of a great interest was a review entitled “PBPK modelling of highly lipid soluble and extracellular solutes” [8], which extensively discussed two classes of drugs, namely the highly lipid soluble (HLS) solutes, and the extracellular (ECS) solutes. These submissions clearly indicate the utilization and significance of PBPK in various stages of drug development.

## References

[1] Shaik A.N.. Changing trends in use of hepatocytes and microsomes for evaluating metabolism studies in drug discovery. ADMET and DMPK 4 (2016) 60-61.

[2] Basu S. Shaik A.N.. Is there a paradigm shift in use of microsomes and hepatocytes in drug discovery and development? ADMET and DMPK 4 (2016) 114-116.

[3] Shaik A.N. Bohnert T. Williams D.A. Gan L.L. LeDuc B.W.. Mechanism of drug-drug interactions between warfarin and statins. Journal of pharmaceutical sciences 105 (2016) 1976-1986. 27103011 10.1016/j.xphs.2016.03.011

[4] Basu S. Shaik A.N.. Role of drug transporters in drug development: a qualitative and quantitative approach. ADMET and DMPK 5 (2017) 57-58.

[5] Grandoni S. Cesari N. Brogin G. Puccini P. Magni P.. Building in-house PBPK modelling tools for oral drug administration from literature information. ADMET and DMPK 7 (2019) 4-21. 10.5599/admet.638 PMC8957249 35350741

[6] Gao G. Law F.C.P. Wong R.N.S. Mak N.K. Yang M.S.. A physiologically based pharmacokinetic model of oseltamivir phosphate and its carboxylate metabolite for rats and humans. ADMET and DMPK 7 (2019) 22-43. 10.5599/admet.628 PMC8957250 35350745

[7] Savoca A. Manca D.. Physiologically-based pharmacokinetic simulations in pharmacotherapy: selection of the optimal administration route for exogenous melatonin. ADMET and DMPK 7 (2019) 44-59. 10.5599/admet.625 PMC8957252 35350746

[8] Levitt D.G.. PKQuest: PBPK modelling of highly lipid soluble and extracellular solutes. ADMET and DMPK 7 (2019) 60-75. 10.5599/admet.579 PMC8957251 35350744

